# Roles of vascular and metabolic components in cognitive dysfunction of Alzheimer disease: short- and long-term modification by non-genetic risk factors

**DOI:** 10.3389/fnagi.2013.00064

**Published:** 2013-11-05

**Authors:** Naoyuki Sato, Ryuichi Morishita

**Affiliations:** ^1^Department of Clinical Gene Therapy, Graduate School of Medicine, Osaka UniversityOsaka, Japan; ^2^Department of Geriatric Medicine, Graduate School of Medicine, Osaka UniversityOsaka, Japan

**Keywords:** diabetes mellitus, hypertension, dyslipidemia, Alzhimer’s disease, abeta, tauopathies

## Abstract

It is well known that a specific set of genetic and non-genetic risk factors contributes to the onset of Alzheimer disease (AD). Non-genetic risk factors include diabetes, hypertension in mid-life, and probably dyslipidemia in mid-life. This review focuses on the vascular and metabolic components of non-genetic risk factors. The mechanisms whereby non-genetic risk factors modify cognitive dysfunction are divided into four components, short- and long-term effects of vascular and metabolic factors. These consist of (1) compromised vascular reactivity, (2) vascular lesions, (3) hypo/hyperglycemia, and (4) exacerbated AD histopathological features, respectively. Vascular factors compromise cerebrovascular reactivity in response to neuronal activity and also cause irreversible vascular lesions. On the other hand, representative short-term effects of metabolic factors on cognitive dysfunction occur due to hypoglycemia or hyperglycemia. Non-genetic risk factors also modify the pathological manifestations of AD in the long-term. Therefore, vascular and metabolic factors contribute to aggravation of cognitive dysfunction in AD through short-term and long-term effects. β-amyloid could be involved in both vascular and metabolic components. It might be beneficial to support treatment in AD patients by appropriate therapeutic management of non-genetic risk factors, considering the contributions of these four elements to the manifestation of cognitive dysfunction in individual patients, though all components are not always present. It should be clarified how these four components interact with each other. To answer this question, a clinical prospective study that follows up clinical features with respect to these four components: (1) functional MRI or SPECT for cerebrovascular reactivity, (2) MRI for ischemic lesions and atrophy, (3) clinical episodes of hypoglycemia and hyperglycemia, (4) amyloid-PET and tau-PET for pathological features of AD, would be required.

## INTRODUCTION

**FIGURE 1:**
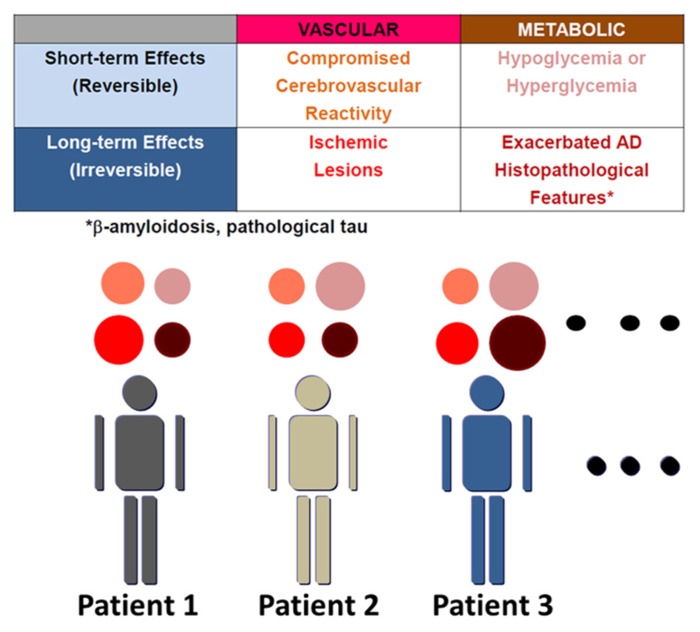
**Variable mechanisms by which AD patients with non-genetic risk factors manifest cognitive dysfunction.** The mechanisms whereby non-genetic risk factors modify cognitive dysfunction are divided into four elements: short- and long-term effects of vascular and metabolic factors. These are (1) compromised vascular reactivity, (2) vascular lesions, (3) hypo/hyperglycemia, and (4) exacerbated AD histopathological features, respectively. AD histopathological features include β-amyloidosis and pathological tau. β-amyloid also compromises vascular reactivity and causes microhemorrhages due to cerebral amyloid angiopathy. The contribution of these four elements to manifestation of cognitive dysfunction varies among patients, though all components must not always be present.

The number of dementia patients is over 30 million worldwide (Dartigues, 2009). Alzheimer disease (AD) accounts for about 50% of cases. Though AD is a progressive neurodegenerative disorder, clinical therapy for this devastating disease is still limited to choline esterase inhibitors and *N*-methyl-D-aspartate activated receptor antagonists. AD is pathologically characterized by cerebral atrophy, particularly of the hippocampus as well as temporal and parietal lobes, and microscopically by senile plaques, neurofibrillary tangles (NFT), and neuronal cell death. β-amyloid (Aβ), a peptide consisting of 38–43 amino acids, was discovered in cerebral amyloid angiopathy (CAA; Glenner and Wong, 1984) and senile plaques. Subsequently, amyloid precursor protein (APP) was cloned based on the Aβ sequence. Familial AD has been found to be caused by mutations in APP and presenilin (Levy-Lahad et al., 1995; Rogaev et al., 1995; Sherrington et al., 1995). Both mutations cause overproduction of Aβ, particularly its longer form, Aβ42, which is more prone to aggregate, although the mechanism whereby presenilin mutations increase Aβ42/Aβ40 ratio is still under investigation (De Strooper et al., 2012; Haass et al., 2012; Sato et al., 2012a). On the other hand, sporadic cases comprise more than 95% of AD. Although some drugs based on the Aβ hypothesis are in clinical trials, the search for alternative therapeutic targets against AD should be encouraged. Such targets could emerge from an understanding of the mechanisms whereby risk factors increase the incidence of sporadic AD. Genetic and non-genetic risk factors contribute to sporadic AD (Fotuhi et al., 2009). APOEε4 is the strongest genetic risk factor for sporadic AD. APOEε4 promotes the development of senile plaques, although its mechanism is yet to be determined. On the other hand, non-genetic risk factors include diabetes (Ott et al., 1999), hypertension (Takeda et al., 2008) and dyslipidemia in mid-life (Shepardson et al., 2011a). However, the mechanisms by which these non-genetic risk factors may modify cognitive function have not been coordinately understood. Here, we summarize these mechanisms by dividing them into four components (**Figure 1**), and propose clinical application of this concept in order to understand the pathogenesis of cognitive dysfunction in individual patients. These are short- and long-term effects of vascular and metabolic factors: (1) compromised vascular reactivity, (2) vascular lesions, (3) hypo/hyperglycemia, and (4) exacerbated AD histopathological features, respectively. Vascular factors compromise cerebrovascular reactivity in response to neuronal activity and also cause irreversible vascular lesions. On the other hand, representative short-term effects of metabolic factors on cognitive function occur due to hypoglycemia and hyperglycemia. Non-genetic risk factors also modify the pathological manifestations of AD in the long-term. Therefore, vascular and metabolic components contribute to aggravation of cognitive dysfunction in AD through short-term and long-term effects.

### HYPERTENSION AND AD

The number of patients with hypertension is now estimated to be approximately 40 million. One half of these patients are untreated, and half of those receiving treatment are poorly controlled (Prince, 1997). Epidemiological studies showed that patients who developed dementia showed an increase in blood pressure from mid-life through to late-life, compared to those who did not develop dementia (Stewart et al., 2009). Pathological investigations also indicate that hypertension causes an increase in white matter lesions (Firbank et al., 2007). Therefore, hypertension causes vascular lesions such as stroke, white matter lesions, and microhemorhages, which might cause cognitive dysfunction. In addition, hypertension might cause functional cerebrovascular abnormalities. Therefore, hypertension mainly modifies AD through vascular factors, though it might have an influence on pathological processes in AD as discussed later.

### DIABETES AND AD

Numerous epidemiological studies have also demonstrated that patients with diabetes have a significantly higher risk of developing AD (Kopf and Frolich, 2009; Maher and Schubert, 2009). However, the mechanism whereby diabetes increases the risk of AD is not fully understood. In the Rotterdam study, diabetes almost doubled the risk of dementia and AD (Ott et al., 1999). In a Japanese cohort, the Hisayama study also indicated that glucose intolerance increased the incidence of AD two to fourfold. Moreover, a meta-analysis of 14 studies also confirmed that diabetes increases the risk of AD (Kopf and Frolich, 2009).

### DYSLIPIDEMIA AND AD

Midlife dyslipidemia could also be a risk factor for AD (Shepardson et al., 2011a), although it is reported that late-life dyslipidemia might be protective against AD (Mielke et al., 2005). Though whether dyslipidemia is a risk for AD is still controversial, it is noteworthy that statins might have preventive effects against AD (Shepardson et al., 2011b). Retrospective cohort studies by Wolozin et al. (2000) and Jick et al. (2000) independently suggested that statin users had a lower prevalence of dementia. However, a randomized controlled study failed to show beneficial effects on the cognitive decline in AD (Feldman et al., 2010). On the other hand, the Rotterdam study, a prospective cohort study, demonstrated that compared to non-statin users and non-statin lipid-lowing drug users, users of both lipophilic and hydrophilic statins had a lower incidence of AD by nearly a half (Haag et al., 2009). Therefore, statins could prevent or delay the onset of AD, but not slow cognitive decline once the disease has set in (Sato et al., 2012b). Several studies have already shown that statins might reduce the Aβ level in the brain (Fassbender et al., 2001; Burns et al., 2006; Ostrowski et al., 2007; Kurinami et al., 2008). Thus, dyslipidemia and use of statins could have opposite influences on cognitive function through different mechanisms.

## SHORT-TERM MODIFICATION BY VASCULAR FACTORS

Cognition is closely related to cerebrovascular function (Iadecola et al., 2009; Dickstein et al., 2010). Short-term modification by vascular factors is mediated through reversible dysfunction of vascular reactivity to neuronal stimulation. Non-genetic risk factors such as hypertension and diabetes compromise vascular reactivity. Hypertension reduces cerebrovascular reactivity in humans (Griffith et al., 1978; Maeda et al., 1994). Hypertension causes deterioration of cerebrovascular function through physical pressure load and angiotensin-mediated signal transduction. On the other hand, it is also well known that diabetic complications involve microangiopathy (Muris et al., 2012). Indeed, diabetes affects vascular reactivity (Caballero et al., 1999; Pasquier et al., 2006). Moreover, our study using animals suggested that diabetes increases Aβ accumulation in the cerebral vasculature (Takeda et al., 2010b). Therefore, diabetes could aggravate vascular reactivity through multiple pathways including hyperglycemia, hyperinsulinemia, and increased Aβ accumulation. In fact, Aβ itself reduces endothelial function *in vitro* (Hayashi et al., 2009) and vascular reactivity in mice (Niwa et al., 2000) and humans (Dumas et al., 2012). Moreover, we confirmed that an angiotensin receptor blocker improved cognitive function and restored cerebrovascular function in an AD mouse model through reduction of Aβ-induced cellular stress (Takeda et al., 2009a). Therefore, vascular factors compromise cerebrovascular function through physical pressure load, osmotic load, angiotensin or insulin signal, and Aβ load.

## LONG-TERM MODIFICATION BY VASCULAR FACTORS

Alzheimer disease with cerebrovascular disease is more common than previously recognized. It is understandable that cerebrovascular lesions aggravate cognitive function in AD patients (Richard and Pasquier, 2012). As hypertension and diabetes increase cerebrovascular lesions, these non-genetic risk factors for AD increase the risk of AD by increasing cerebrovascular lesions as well.

### HYPERTENSION AND VASCULAR LESIONS

It has been indicated that mid-life hypertension is a risk factor for the development of AD (Skoog et al., 1996; Launer et al., 2000; Kivipelto et al., 2001; Takeda et al., 2008). As hypertension increases cerebrovascular necrosis and arteriosclerosis, antihypertensive therapies could suppress cognitive function decline (Peters et al., 2008; Takeda et al., 2008). In the Syst-Eur study, a randomized controlled study, antihypertensive medication, which included nitrendipine, enalapril and hydrochlorothiazide, in elderly hypertensive patients decreased the onset risk not only for vascular dementia, but also for AD (Forette et al., 2002). In addition, SCOPE, Study on Cognition and Prognosis in the Elderly, found that candesartan, an angiotensin receptor blocker, inhibited cognitive deterioration in patients with mild cognitive impairment (Skoog et al., 2005). A recent meta-analysis of studies including HYVET-cog (Hypertension in the Very Elderly Trial cognitive function assessment) indicated that the occurrence of dementia is significantly reduced by antihypertensive treatment (Peters et al., 2008). To determine whether antihypertensive therapy can prevent dementia requires a study setting dementia prevention as a primary endpoint. The OSCAR study (Observational Study on Cognitive function And systolic blood pressure Reduction) was conducted to confirm whether hypertensive patients treated with eprosartan show improvement in MMSE score (Shlyakhto, 2007). Results from a subgroup of OSCAR are supportive of the hypothesis that this treatment may be associated with preservation of cognitive function (Radaideh et al., 2011). Another report from OSCAR of a retrospective investigation suggested that blood pressure responses after treatment coincided with stabilization of MMSE in difficult-to-treat hypertensive patients (Petrella et al., 2012). Therefore, further clinical studies are warranted to clarify whether antihypertensive drugs could prevent dementia and inhibit progression of the disease.

### DIABETES AND VASCULAR LESIONS

Diabetes also increases cerebrovascular lesions (van Elderen et al., 2010), which aggravates cognitive dysfunction in AD. To understand the mechanism whereby diabetes increases the risk of AD, we generated AD model mice with a diabetic phenotype by crossbreeding APP Tg mice and leptin-deficient *ob/ob* mice. We examined Aβ burden in the cerebral vessels in APP^+^-*ob*/*ob* and found that these mice had more severe CAA than did single APP Tg mice (Takeda et al., 2010b). CAA is one of the major characteristics observed in AD and vascular aging. CAA triggers hemorrhagic (Greenberg and Vonsattel, 1997) and contributes to the clinical presentation of dementia (Pfeifer et al., 2002). We also found that APP^+^-*ob*/*ob* mice showed up-regulation of RAGE, the receptor for AGE (Brownlee et al., 1988), in the vasculature. It is reported that RAGE mediates amplification of inflammatory responses (Basta et al., 2002). Indeed, inflammatory cytokines such as IL-6 and TNFα were upregulated around the cerebrovasculature in APP^+^-*ob*/*ob *(Takeda et al., 2010b). Therefore, diabetes also could affect cerebrovascular vascular lesions through increased expression of RAGE and subsequent chronic inflammation.

### STATINS AND VASCULAR LESIONS

Dyslipidemia is also a risk factor for vascular disease, especially cardiovascular disease. Whether dyslipidemia is a risk for cerebrovascular disease is relatively unclear, probably because the power of dyslipidemia to promote cardiovascular disease is too strong. Anti-dyslipidemia statins are protective against vascular change. In clinical studies, statins have been shown to prevent secondary stroke (Ni Chroinin et al., 2013). Several published studies, including ours, demonstrated that statins restored cognitive function after experimental stroke through their pleiotropic effects (Shimamura et al., 2007; Mayanagi et al., 2008). Because hypertension and diabetes, in addition to dyslipidemia, increase the risk of stroke, statins could prevent worsening of cognitive dysfunction in AD patients with these common diseases.

## SHORT-TERM MODIFICATION BY METABOLIC FACTORS

Diabetic patients experience hyperglycemia or hypoglycemia during dietary and drug control of plasma glucose levels. Both these conditions have an influence on patients’ cognitive dysfunction. Because the brain uses mainly glucose as an energy source, hypoglycaemia causes defects of neuronal function, though lactate can also be used in this situation (Rasmussen et al., 2011; Wyss et al., 2011). Failure of neuronal networking including cholinergic and GABAergic pathways also might contribute to cognitive impairment in a hypoglycemic state (Sherin et al., 2012). On the other hand, hyperglycemia also compromises cognitive dysfunction, due to ketoacidosis and a hyperglycemic hyperosmotic state. Cognitive dysfunction due to hyperglycemia or hypoglycemia is reversible. However, repeated episodes of severe hypoglycemia are reported to also be a risk for the development of dementia (Whitmer et al., 2009).

## LONG-TERM MODIFICATION BY METABOLIC FACTORS

### HYPERTENSION AND AD PATHOLOGY

Hypertension could possibly modify AD risk by increasing the pathological progression of AD in addition to ischemic lesions. In a study of autopsied brain, the incidence of senile plaques and NFT in hypertensive patients was approximately 2 and 4 times higher than control, respectively (Sparks et al., 1995). Interestingly, it is reported that valsartan, an angiotension receptor blocker, improved cognitive deterioration in AD model mice through anti-Aβ effects; that is, inhibition of Aβ oligomerization and promotion of Aβ degradation by an insulin-degrading enzyme (Wang et al., 2007). However, the mechanism by which hypertension increases AD risk needs further investigation because the underlying mechanisms are not so straightforward.

### DIABETES AND Aβ

Epidemiological and neuropathological studies have provided many important insights into the mechanism whereby diabetes increases the risk of AD. A cohort study indicated that insulin resistance in mid-life is associated with the development of senile plaques in later life (Matsuzaki et al., 2010). In contrast, retrospective studies suggested that the magnitude of senile plaques and NFT was comparable between AD patients with and without diabetes (Kalaria, 2009). These results seem to contradict each other. Several groups reported that a high-fat diet caused Aβ accumulation in the brain of wild type rabbits (Sparks et al., 1994) and APP Tg mice. There are several proposed mechanisms for this phenomenon, such as compromised autophagy in an insulin-resistant condition (Son et al., 2012) and upregulation of BACE1, which cleaves APP, in a diabetic condition (Guglielmotto et al., 2012). Although feeding a high-fat diet caused severe memory deficit in APP Tg with NSY background (Nagoya-Shibata-Yasuda) mice, which are established as an inbred animal model with spontaneous development of diabetes (Shibata and Yasuda, 1980; Ueda et al., 2000), we observed no increase in brain Aβ load by a high-fat diet (Takeda et al., 2010b). Similarly, we found no difference in brain Aβ accumulation between APP^+^-*ob*/*ob* and APP mice without diabetes (Takeda et al., 2010b). These findings as to whether diabetes increases Aβ accumulation in the AD mouse brain seem to be inconsistent. The magnitude of inflammation evoked by diabetes, which activates microglia to clear Aβ, might be involved in this inconsistency.

### DIABETES AND BRAIN INSULIN SIGNALING

Insulin signaling might be impaired in the AD and diabetic brain. Insulin receptors are ubiquitous in the cortex and hippocampus (Havrankova et al., 1978; Hill et al., 1986; Wickelgren, 1998), and circulating insulin accesses the brain by crossing the blood–brain barrier (Banks, 2004). In the advanced AD brain, the levels of insulin and IGF (insulin-like growth factor)-1 were significantly reduced relative to controls (Rivera et al., 2005). After insulin binds to the insulin receptor, which activates its tyrosine kinase, IRS-1 protein undergoes tyrosine phosphorylation and binds phosphatidylinositol 3-kinase (PI3K; Sun et al., 1991), which activates AKT and glycogen-synthase kinase-3β (GSK3β; Sutherland et al., 1993; Cross et al., 1995; Neumann et al., 2008). *In vitro*, Aβ increases tau phosphorylation through AKT-GSK3β (Tokutake et al., 2012). Consistent with this result, the AD brain manifested reduced responses to insulin and IGF-1 signaling (Talbot et al., 2012). The levels and activities of the insulin-PI3K-AKT pathway were also reported to be decreased in AD and diabetic brains (Liu et al., 2011). Consistent with these human studies, our APP^+^-*ob*/*ob* mice also manifested impaired insulin signaling in the brain (Takeda et al., 2010b). These results raise the possibility that impaired insulin signaling might be involved in the pathogenesis of AD with or without diabetes (Sato et al., 2011; Takeda et al., 2011). Similarly, IGF-1, IGF-2 and their receptors also exist in the CNS (Shemer et al., 1987; Rotwein et al., 1988; Araujo et al., 1989; Chen et al., 2011). Importantly, brain-specific insulin receptor knockout mice showed no alteration in the brain (Schubert et al., 2004), suggesting compensation of IGF receptor signaling for insulin signaling. Taken together, these findings indicate that insulin/IGF signaling might be impaired in the AD with diabetes brain, and this signaling might have an impact on aging and disease-related brain dysfunction.

### INSULIN SIGNALING AND Aβ

The next question is whether impaired insulin signaling has any influence on Aβ metabolism. *In vitro* studies suggested that insulin/IGF signaling is involved in Aβ generation, clearance, and trafficking (Gasparini et al., 2001; Carro et al., 2006; Freude et al., 2009b). While soluble Aβ oligomers and Aβ aggregates are in equilibrium (Sato et al., 2006), reduced IGF signaling is reported to decrease soluble Aβ oligomers, increasing Aβ aggregates (Cohen et al., 2009). In contrast, another group reported that a reduction of IGF signaling decreased Aβ deposition, suggesting an opposite shift (Freude et al., 2009a). Similarly, loss of a downstream mediator of insulin/IGF signaling, IRS (insulin receptor substrate)-2, is reported to reduce Aβ deposition (Freude et al., 2009a; Killick et al., 2009). Moreover, it is also reported that suppression of the insulin receptor also decreased Aβ deposition (Murakami et al., 2011). Our APP^+^-*ob*/*ob* mice manifested a reduction in insulin signaling with no change in brain Aβ level, but increased Aβ deposition in the cerebral vasculature (Takeda et al., 2010b). Therefore, although the effects of insulin signaling on Aβ metabolism are not so simple, we speculate that reduced insulin signaling might affect control of protein quality and quantity in diabetic AD mice.

### DIABETES AND TAU

Diabetes could also exacerbate tau phosphorylation and formation of NFT. Although tau physiologically promotes the assembly and stabilization of microtubules, hyperphosphorylated tau sequesters normal tau, and disrupts microtubules (Iqbal et al., 1994, 2009). Retrospective clinicopathological studies suggested that the magnitude of NFT is comparable in AD with and without diabetes (Kalaria, 2009), though a retrospective study might reflect the features at the end stage of the disease. On the other hand, many groups reported that diabetes increased tau phosphorylation in mouse models. Importantly, in the human diabetic brain, tau phosphorylation is increased at the same sites as hyperphosphorylated in AD (Liu et al., 2009). These studies indicate that diabetes could increase tau phosphorylation, leading to the development of NFT.

### INSULIN SIGNALING AND TAU PHOSPHORYLATION

Indeed, impaired insulin signaling could cause tau phosphorylation. As mentioned above, insulin signaling is well known to be mediated through the PI3K-AKT pathway, with downstream involvement of GSK3β (Sutherland et al., 1993; Cross et al., 1995; Neumann et al., 2008). Because GSK3β phosphorylates tau, insulin inhibits tau phosphorylation in neurons through the inhibition of GSK3β via AKT (Hong and Lee, 1997). In contrast, loss of insulin (Schechter et al., 2005), insulin receptor (Schubert et al., 2004), or IRS-2 (Schubert et al., 2003; Freude et al., 2009a; Killick et al., 2009) results in an increase of tau phosphorylation. These findings indicate that impaired insulin signaling might increase tau phosphorylation. In general, protein phosphorylation is also regulated by phosphatases. Tau is reported to be dephosphorylated by protein phosphatase 2A (Sontag et al., 1996). Moreover, disruption of IRS-2 also downregulates protein phosphatase 2A (Sontag et al., 1996). Therefore, impaired insulin signaling might cause tau phosphorylation by influencing both kinases and phosphatases. Taken together, these findings indicate it is possible that diabetes could promote tau phosphorylation via impaired insulin signaling in the brain.

### MODIFICATION OF DIABETIC PHENOTYPE BY AD

It is also noteworthy that AD could affect diabetic phenotype. Several clinical reports have suggested that AD patients manifest glucose intolerance (Bucht et al., 1983; Meneilly and Hill, 1993; Janson et al., 2004). Consistent with these clinical observations, we found that AD aggravated the diabetic phenotype in two different lines of APP Tg mice with diabetes (Takeda et al., 2010b; Sato et al., 2011). We can speculate on the mechanisms whereby AD affects the diabetic phenotype. First, AD could compromise central control of peripheral glucose metabolism (van de Nes et al., 1998), as recent evidence suggests an important role of the central nervous system in control of glucose homeostasis (Demuro and Obici, 2006; Prodi and Obici, 2006). Second, plasma Aβ could mediate peripheral insulin resistance. We previously reported that plasma Aβ level increases after glucose loading in AD transgenic mice (Takeda et al., 2009b, 2010a), and could change after oral glucose loading in AD patients (Takeda et al., 2012). Therefore, increased plasma Aβ might affect insulin signaling directly in peripheral tissues (Sato and Morishita, 2013; Zhang et al., 2013). Third, Aβ accumulation could occur in the pancreas (Miklossy et al., 2008) and skeletal muscle (Roher et al., 2009), thereby impairing insulin secretion and insulin resistance, respectively. In clinical settings, AD patients might have worse glucose control because they cannot take medication and eat properly. Indeed, poor cognitive function also increases the risk of severe hypoglycemia in patients with type 2 diabetes (Punthakee et al., 2012).

### EFFECT OF STATINS ON Aβ PRODUCTION

As mentioned above, anti-dyslipidemia drugs, statins, might have a preventive effect against AD. We investigated the mechanisms responsible for the reduction of Aβ in the brain by statins. First, Aβ reduction is associated with a reduction in the carboxyl terminal fragment of APP (APP-CTF; Shinohara et al., 2010). Statins reduce the brain Aβ level through increasing APP-CTF trafficking by inhibition of protein isoprenylation. In general, Aβ level is balanced between its production and clearance. We also found that statins reduce brain Aβ level through enhanced Aβ clearance mediated by up-regulation of LRP-1 (LDL receptor related protein-1) expression. Therefore, we can expect an additional effect of brain Aβ reduction by statins to decrease vascular events. There are reports suggesting that statins might transiently and reversibly impair cognitive function (Orsi et al., 2001; King et al., 2003; Wagstaff et al., 2003), especially if the drug is firstly administered to treat patients aged over 75 years. Despite these several reports of statin-associated cognitive impairment, this adverse effect remains a rare occurrence (Rojas-Fernandez and Cameron, 2012). Considering the beneficial effects, statins should be used with close attention to the emergence of adverse effects in elderly patients. Recent PET studies of amyloid confirmed that Aβ begins to accumulate in the brain one or two decades before the manifestation of memory impairment in AD. Statins in mid-life might prevent the onset of AD by reducing Aβ production by enhancing APP-CTF degradation and up-regulating Aβ clearance in the brain. There are over 30,000,000 patients taking statins, and they might benefit from Aβ reduction in the brain in addition to the cholesterol-lowering effect (Sato et al., 2012b).

## CONCLUSION AND PERSPECTIVE

Non-genetic risk factors, such as diabetes, hypertension, and dyslipidemia, modify cognitive dysfunction in AD. The mechanisms of these consequences are divided into four components. These are short- and long-term effects of vascular and metabolic factors: (1) compromised vascular reactivity, (2) vascular lesions, (3) hypo/hyperglycemia, and (4) exacerbated AD histopathological features, respectively. β-amyloid could be involved in both vascular and metabolic components. It might be beneficial to support treatment in AD patients by appropriate therapeutic management of non-genetic risk factors, considering the contributions of these four elements to the manifestation of cognitive dysfunction in individual patients, though all components may not always be present. It should be clarified how these four components interact with each other. To answer this question, a clinical prospective study that follows up clinical features with respect to these four components: (1) functional MRI or SPECT for cerebrovascular reactivity, (2) MRI for ischemic lesions and atrophy, (3) clinical episodes of hypoglycemia and hyperglycemia, and (4) amyloid-PET and tau-PET for pathological features of AD, would be required. Understanding the interaction of the four components will help to elucidate the role of vascular and metabolic factors in cognitive dysfunction of AD and provide beneficial knowledge for the treatment of AD patients with or even without non-genetic risk factors.

## Conflict of Interest Statement

The authors declare that the research was conducted in the absence of any commercial or financial relationships that could be construed as a potential conflict of interest.

## ABBREVIATIONS

APP: amyloid precursor protein
CAA: cerebral amyloid angiopathy
IGF-1: insulin-like growth factor-1
IRS-2: insulin receptor substrate-2
LRP-1: LDL receptor related protein-1
NSY: Nagoya-Shibata-Yasuda mouse
RAGE: receptor for advanced glycation end products
